# Comparative *in vitro* efficacy of aztreonam-avibactam and other alternatives (cefepime-taniborbactam, cefepime-zidebactam, and cefiderocol) against metallo-β-lactamase-producing Enterobacterales isolated in 2024 in France

**DOI:** 10.1128/aac.00176-26

**Published:** 2026-03-27

**Authors:** Cécile Emeraud, Marion Dutkiewicz, Laurent Dortet

**Affiliations:** 1 Team "Resist" UMR1184 Immunology of Viral, Auto-Immune, Hematological and Bacterial Diseases (IMVA-HB), INSERM, Université Paris-Saclay, CEA, LabEx LERMIT, Faculty of Medicine, Le Kremlin-Bicêtre, France; 2 Bacteriology-Hygiene Unit, Bicêtre Hospital, Assistance Publique-Hôpitaux de Paris (APHP) 537860 , Le Kremlin-Bicêtre, France; 3 Associated French National Reference Center for Antibiotic Resistance: Carbapenemase-Producing Enterobacteriaceae, Le Kremlin-Bicêtre, France; 4 SEPSIS Comprehensive Center–IHU SEPSIS, Garches, France; Universita degli Studi di Roma La Sapienza, Rome, Italy

**Keywords:** aztreonam-avibactam, cefiderocol, MBL-producing Enterobacterales, NDM, VIM, antimicrobial resistance, susceptibility

## Abstract

We evaluated the *in vitro* activity of aztreonam-avibactam and other agents against 593 metallo-β-lactamase (MBL)-producing Enterobacterales collected in France in 2024. Aztreonam-avibactam showed the highest activity (98% susceptible), with resistance mainly observed in NDM-5-producing *Escherichia coli* belonging to ST167, ST405, and ST361. Cefepime-zidebactam also exhibited strong activity (96.6% of susceptibility). Cefepime-taniborbactam and cefiderocol showed reduced overall activity (76.4% and 74.9% susceptibility, respectively), with reduced efficacy mainly observed among NDM-producing isolates. Comparison with 1,465 isolates from 2023 showed no increase in aztreonam-avibactam resistance.

## INTRODUCTION

The rapid global dissemination of metallo-β-lactamase (MBL)-producing Enterobacterales, mainly driven by NDM and VIM enzymes, represents a major therapeutic challenge due to limited treatment options.

Among currently available or late-stage agents, aztreonam-avibactam and cefiderocol remain the main therapeutic options, while newer combinations such as cefepime-zidebactam and cefepime-taniborbactam are under development (1). Cefiderocol and cefepime-taniborbactam have demonstrated good activity against VIM-producing isolates, whereas variable efficacy has been reported against NDM producers (2). In contrast, aztreonam-avibactam and cefepime-zidebactam remain highly active against both VIM- and NDM-producing Enterobacterales (1).

Recent reports have described the emergence of reduced susceptibility or resistance to both aztreonam-avibactam and cefiderocol among MBL-producing Enterobacterales, mainly in NDM-producing *Escherichia coli* belonging to high-risk lineages, such as NDM-5 producing *E. coli* ST405 or ST167 (3–7). For both agents, resistance has been associated with similar genetic backgrounds, including alterations in penicillin-binding protein 3 (PBP3), particularly amino-acid insertions such as YRIN or YRIK in the PBP3 sequence, often combined with the acquisition of specific CMY variants (3, 5, 7, 8). Given the rapid evolution of these resistance mechanisms, it is crucial to continuously monitor their activity against circulating clones. In this context, we evaluated the *in vitro* activity of aztreonam-avibactam and other last-resort agents against MBL-producing Enterobacterales isolated in France in 2024.

All non-duplicate MBL-producing Enterobacterales from clinical samples and referred to the French-National Reference Center for Antimicrobial Resistance between May 2024 and January 2025 were included in this study (*n* = 593) (Table S1). Most isolates produced NDM enzymes (*n* = 450), followed by VIM producers (*n* = 88), with a minority of isolates co-producing NDM and OXA-48-like or IMP enzymes. Carbapenemase variants and sequence types were determined by WGS using Illumina technology, as previously described (9). Reads were assembled using Shovill v1.1.0 and SPAdes v3.14.0. MLST and resistome analyses were carried out using the pubMLST and ResFinder. Clonal relatedness was assessed by single-nucleotide polymorphism (SNP) analysis using Snippy v4.6.0.

MICs were determined by broth microdilution using customized Sensititre plates (Thermo Fisher Scientific), with a standardized final inoculum prepared according to the manufacturer’s recommendations. Cefiderocol MICs were determined using UMIC Cefiderocol panels (Bruker Daltonics). All results were interpreted according to EUCAST 2024 breakpoints.

**Fig 1:**
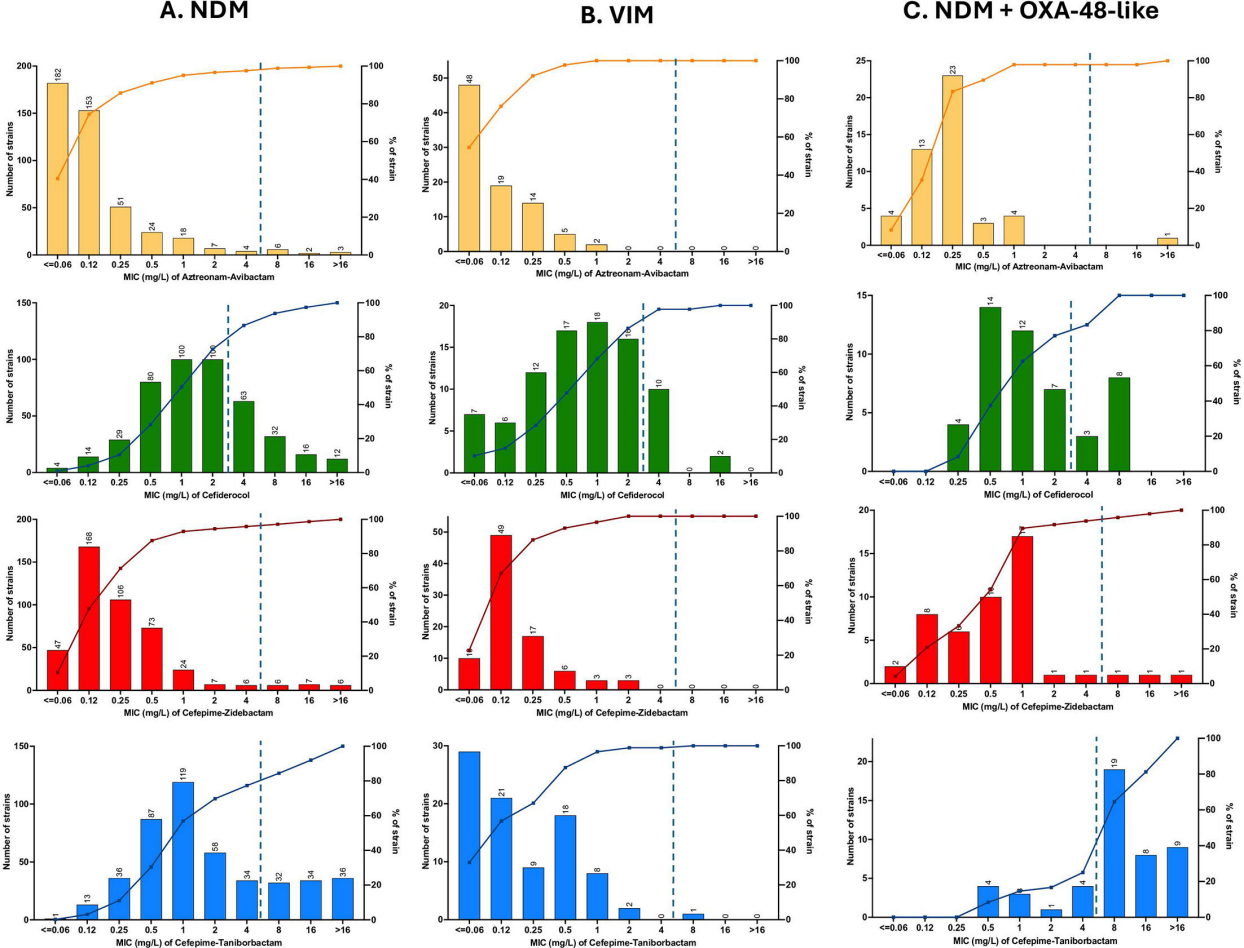
MIC distributions of last-resort antibiotics against MBL-producing Enterobacterales: Aztreonam-avibactam (yellow), cefiderocol (green), cefepime-zidebactam (red), and cefepime-taniborbactam (blue) for isolates producing NDM (**A**), VIM (**B**), or co-producing NDM and OXA-48-like enzymes (**C**).

Aztreonam-avibactam was the most active agent tested, with the lowest MIC, with an overall susceptibility rate of 98.0% and a MIC_90_ of 0.5 mg/L, regardless of MBL type (Table 1, Fig. 1). The 12 resistant strains included nine NDM-5-producing *E. coli* isolates belonging to ST405 (*n* = 5), ST361 (*n* = 3), and ST1284 (*n* = 1), as well as one NDM-35-producing *E. coli* ST2851, one *E. coli* ST2851 co-producing NDM-5 and OXA-181, and one NDM-1-producing *Enterobacter cloacae* complex of ST45. Among these 12 resistant isolates, six co-produced a CMY-type β-lactamase: three not clonally related (249–2,055 SNPs, Table S2) NDM-5-producing *E. coli* ST405 produced CMY-42, one NDM-35-producing *E. coli* ST2851 produced CMY-145, and two independent (680 SNPs between the two strains) NDM-5-producing *E. coli* ST361 produced CMY-145. Notably, all 11 resistant *E. coli* isolates harbored a YRIN or YRIK insertion in PBP3. These findings are consistent with previous reports linking aztreonam-avibactam resistance to NDM-5 production and specific *E. coli* sequence types (3, 7, 10). Notably, aztreonam-avibactam-resistant isolates frequently displayed elevated MICs to cefiderocol and cefepime-taniborbactam, whereas susceptibility to cefepime-zidebactam was preserved (Table S1).

**TABLE 1:** EUCAST breakpoints, MIC_50_, MIC_90_, and susceptibility rates for antibiotics tested against 593 MBL-producing Enterobacterales^*b*^^,^^*c*^

Antibiotics	Breakpoint EUCAST (mg/L)		Total MBLs (*n* = 593)					NDM (*n* = 450)					VIM (*n* = 88)					NDM + OXA–48–like (*n* = 48)				
	S ≤	R >	MIC_50_ (mg/L)	MIC_90_ (mg/L)	%S	%I	%R	MIC_50_ (mg/L)	MIC_90_ (mg/L)	%S	%I	%R	MIC_50_ (mg/L)	MIC_90_ (mg/L)	%S	%I	%R	MIC_50_ (mg/L)	MIC_90_ (mg/L)	%S	%I	%R
Aztreonam (ATM)	**1**	**4**	>16	>16	19.1%	4.8%	76.1%	>16	>16	14.9%	7.9%%	77.2%	2	>16	45.5%	13.9%	40.9%	>16	>16	8.3%	4.2%	87.5%
Aztreonam-avibactam (AZA)	**4**	**4**	**0.12**	**0.5**	98.00%	**–^*d*^**	2.0%	**0.12**	**0.5**	97.56%	**–**	2.4%	**≤0.06**	**0.25**	100%	**–**	0%	**0.25**	**0.5**	97.9%	**–**	2.1%
Cefepime (FEP)	**1**	**4**	>16	>16	1.2%	10.6%	88.2%	>16	>16	0.2%	4.9%%	94.9%	8	>16	18.2%	32.9%	48.9%	>16	>16	0%	0%	100%
Cefepime-enmetazobactam (FEN)	**4**	**4**	>16	>16	9.3%	**–**	90.7%	>16	>16	3.3%	**–**	96.7%	8	>16	44.3%	**–**	55.7%	>16	>16	0%	**–**	100%
Cefepime-taniborbactam (FTA)	**4** ^*a*^	**4** ^*a*^	**1**	16	76.4%	**–**	23.6%	**1**	16	77.3%	**–**	22.7%	**0.12**	**1**	98%	**–**	1.1%	8	>16	25%	**–**	75%
Cefepime-zidebactam (FEZ)	**4** ^*a*^	**4** ^*a*^	**0.25**	**1**	96.3%	**–**	3.7%	**0.25**	**1**	95.8%	**–**	4.2%	**0.12**	**0.5**	100%	**–**	0%	**0.5**	**1**	93.7%	**–**	6.3%
Cefiderocol (FDC)	**2**	**2**	**1**	8	74.9%	**–**	25.1%	**1**	8	72.7%	**–**	27.3%	**1**	4	86.4%	**–**	13.6%	**1**	8	77.1%	**–**	22.9%
Temocillin (TEM)	**8**	**16**	64	>128	11.8%	35.9%	52.3%	32	>128	13.6%	43.5%	42.9%	64	>128	19.3%	9.1%	71.6%	>128	>128	0%	0%	100%
Amikacin (AMK)	**8**	**8**	**4**	>16	60.9%	–	39.1%	**4**	>16	59.1%	–	40.9%	**4**	**8**	**90.9%**	–	9.1%	>16	>16	20.8%	–	79.2%

^*a*^ Unofficial breakpoint, cefepime high-dose breakpoint was used.

^*b*^ Breakpoint only for *E. coli* and *C. koseri*.

^*c*^ Bold values indicate MIC50 and MIC90 values within the susceptible range.

^*d*^ –, no intermediate category defined for this antibiotic.

Cefiderocol retained activity against most isolates, with an overall susceptibility rate of 74.9% and a MIC_90_ of 8 mg/L. VIM-producing isolates were more susceptible than NDM producers (86.4% vs 72.7%), while susceptibility among NDM + OXA-48-like co-producers reached 77.1%. Cefiderocol MIC distributions differed by carbapenemase type, with slightly lower MICs for VIM producers compared to NDM producers, while NDM + OXA-48-like co-producers showed similar profiles to NDM isolates (Fig. 1). Thirty-one isolates exhibited high-level resistance to cefiderocol (MIC ≥ 16 mg/L), mainly NDM producers, including NDM-5 (*n* = 16), NDM-1 (*n* = 8), and NDM-7 (*n* = 4). Most of these strains were *E. coli* (*n* = 15) and *E. cloacae* complex (*n* = 9) isolates. In both species, a wide clonal diversity was observed, with multiple sequence types represented. The only recurrent lineage was NDM-5-producing *E. coli* ST167, identified in 7 of the 15 highly resistant *E. coli* isolates, which were not clonally related (120–6,054 SNPs; Table S2). These findings are consistent with previous reports of reduced cefiderocol susceptibility among NDM-producing isolates (11). Notably, among the 63 cefiderocol-resistant *E. coli*, 49 harbored a YRIN or YRIK insertion in PBP3, and 16 showed a frameshift in *cirA* (5).

Among the newer cefepime/β-lactamase inhibitor combinations, cefepime-zidebactam showed strong activity, with an overall susceptibility rate of 96.3% and consistently low MICs across all MBL types, including isolates co-producing NDM and OXA-48-like enzymes (Table 1, Fig. 1). Among the 22 isolates resistant to cefepime-zidebactam, 3 belonged to *Providencia* spp. or *Serratia* spp., which are intrinsically less susceptible to this combination, 4 were *E. coli*, and 15 were *Klebsiella pneumoniae*, all producing NDM enzymes. Among the *K. pneumoniae* isolates, seven were NDM-14-producing ST147, a clone recently emerging in France (12), and were clonally related, differing by 6–37 SNPs (Table S2). Among these resistant isolates, only one *E. coli* strain harbored a mutation in the PBP2-encoding gene *mrdA* (N571K), and three *K. pneumoniae* isolates carried a T331P substitution.

In contrast, cefepime-taniborbactam exhibited variable activity depending on carbapenemase type (Fig. 1). While susceptibility reached 98% among VIM producers, it decreased to 77.3% among NDM producers and to 25% among NDM + OXA-48-like co-producers (Table 1). Overall, 88 isolates (14.8%) displayed high-level resistance to cefepime-taniborbactam (MIC ≥ 16 mg/L), all of which were NDM producers. High-level resistance was more frequent among *E. coli* (57%) than *K. pneumoniae* (11%). Furthermore, NDM-5 producers were more frequently resistant than those producing NDM-1, with 45% of NDM-5-producing isolates showing MICs ≥ 16 mg/L versus 4% for those producing NDM-1. The most prevalent highly resistant clones to cefepime-taniborbactam were NDM-5-producing *E. coli* ST410 (20/88, 22.7%), with no clonal relatedness (17–1,893 SNPs; Table S2) and *K. pneumoniae* ST147 producing various NDM variants (14/88, 15.9%).

Taken together, these comparative results highlight distinct activity profiles among last-resort agents tested against MBL-producing Enterobacterales. While aztreonam-avibactam and cefepime-zidebactam maintained low MICs across most genetic backgrounds, cefiderocol and cefepime-taniborbactam displayed more heterogeneous activity, particularly among NDM-producing isolates. Importantly, resistance to one agent did not systematically predict resistance to all others, emphasizing the need for individual susceptibility testing rather than reliance on class-based assumptions. This observation is particularly relevant in settings with a high prevalence of NDM-producing *E. coli*, where therapeutic options may be further constrained.

**Fig 2:**
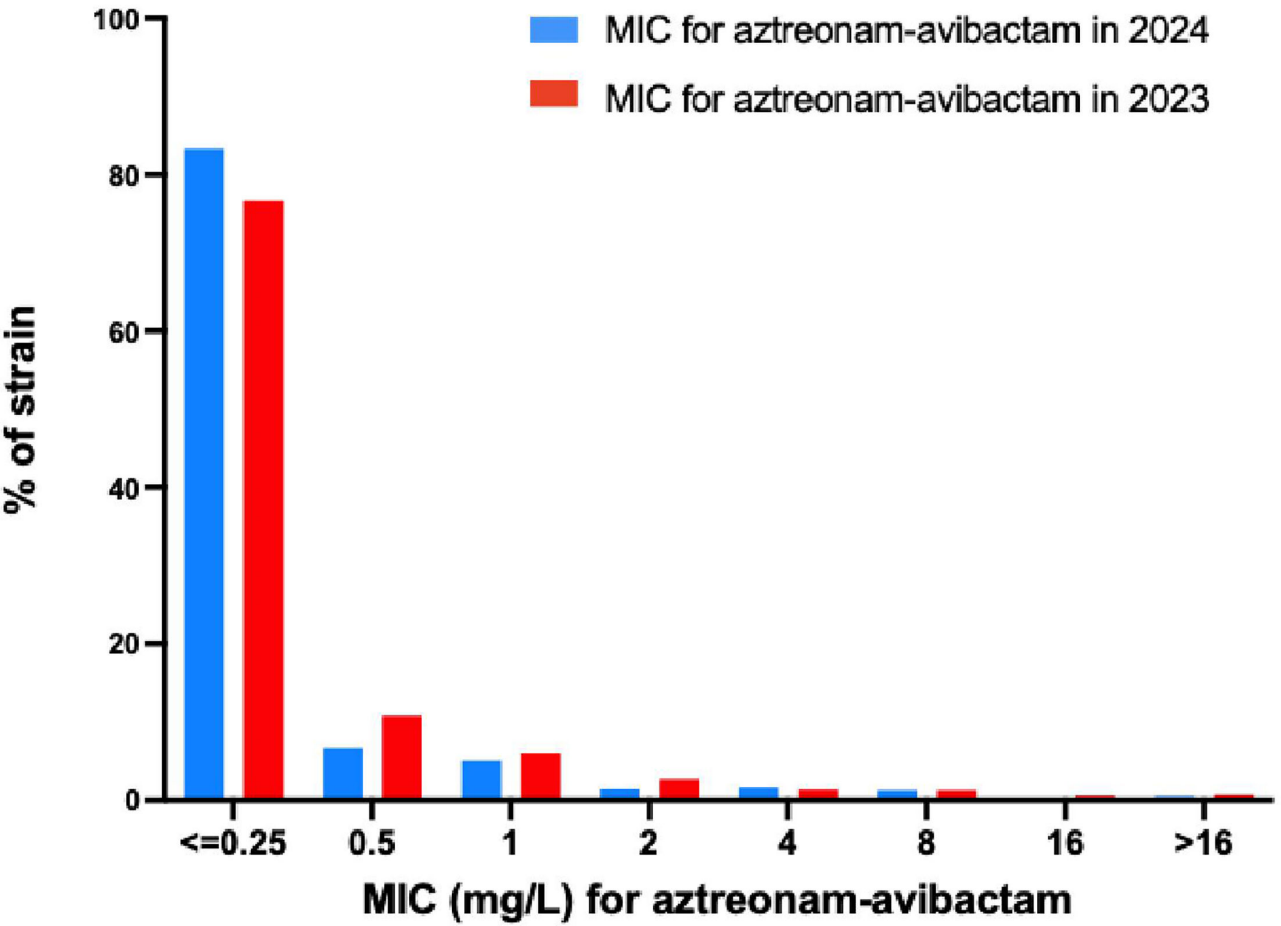
Comparison of aztreonam-avibactam MIC distributions among MBL-producing Enterobacterales received at the F-NRC in 2023 and 2024. MIC distributions are shown for isolates collected in 2023 (red) and 2024 (blue).

To evaluate potential changes in susceptibility over time, we compared MIC distributions of MBL-producing isolates collected in 2023 (*n* = 1,465) and in 2024 (*n* = 1,249) for aztreonam-avibactam. These collections included primarily *K. pneumoniae* (28.4% in 2023 and 33.6% in 2024), *E. coli* (28.4% in 2023 and 23.4% in 2024), *E. cloacae* complex (24% in 2023 and 26.4% in 2024), and *C. freundii* (13% in 2023 and 11.5% in 2024). Regarding carbapenemase variants, most of the isolates produced NDM-1 (37.3% in 2023 and 39.6% in 2024), NDM-5 (33.2% in 2023 and 31.9% in 2024), VIM-1 (8.7% in 2023 and 10.1% in 2024), or VIM-4 (8% in 2023 and 5.3% in 2024). A statistically significant difference in MIC distributions between 2023 and 2024 was observed (*P* = 4.6 × 10⁻⁵, Mann-Whitney *U*-test), with a higher proportion of isolates exhibiting lower MICs in 2024 compared to 2023 (Fig. 2).

In conclusion, this national surveillance study confirms that aztreonam-avibactam remains the most active option against MBL-producing Enterobacterales in France, with sustained activity over time. Cefepime-zidebactam demonstrated excellent *in vitro* activity across all MBL types and represents a promising alternative once available. Cefiderocol retained activity against most isolates but showed lower susceptibility rates among NDM producers, which, together with ongoing challenges in susceptibility testing, may limit its use as empirical therapy.

## SUPPLEMENTAL MATERIAL

Table S1 aac.00176-26-s0001.xlsx Epidemiological, microbiological, and antimicrobial susceptibility characteristics of the 593 MBL-producing Enterobacterales isolates included in this study.

ASM does not own the copyrights to Supplemental Material that may be linked to, or accessed through, an article. The authors have granted ASM a non-exclusive, world-wide license to publish the Supplemental Material files. Please contact the corresponding author directly for reuse.
